# The Uniform System of Accounts for Hospitals and Institutions

**Published:** 1903-11-28

**Authors:** 


### EDITOR'S LETTER-BOX.

**[Our Correspondents are reminded that prolixity is a great bar to publication, and that brevity of style and conciseness of statement greatly facilitate early insertion.]**

### THE UNIFORM SYSTEM OF ACCOUNTS FOR HOSPITALS AND INSTITUTIONS.

Mr. A. P. Payne, secretary of the Brisbane Hospital, writes under date Brisbane, October 17th, 1903: Some time ago I wrote you suggesting that, for the advancement of the cause, credit should be given to the institutions that were loyal to the uniform system of accounts.

In this connection I desire to acknowledge gratefully the generous response that you have made. . . .

I still think, although you should perhaps be the better judge, that more leverage might be applied to the laggards if you inserted a page setting forth prominently (in column) the names of the hospitals that exhibit faithful adherence to the system. This would catch the eye of the press, who would prod up the recalcitrants. In this city there is only one hospital (this one) that fully applies the system, and although it is a Government requirement in all the Queensland hospitals *the three Government institutions in the State only apply it to a very limited extent*, and for all the opportunity of comparison they afford these might almost as well leave it alone.

I have been advocating the Burdett system in season and out of season for some years, with meagre result. There is no conservatism harder to overcome than lazinesss.

